# The Essential Role of anxA2 in Langerhans Cell Birbeck Granules Formation

**DOI:** 10.3390/cells9040974

**Published:** 2020-04-15

**Authors:** Shantae M. Thornton, Varsha D. Samararatne, Joseph G. Skeate, Christopher Buser, Kim P. Lühen, Julia R. Taylor, Diane M. Da Silva, W. Martin Kast

**Affiliations:** 1 Department of Molecular Microbiology and Immunology, University of Southern California, Los Angeles, CA 90033, USA; shantae14@gmail.com (S.M.T.); varsha2030@yahoo.com (V.D.S.); skeate@med.usc.edu (J.G.S.); jrtaylor924@gmail.com (J.R.T.); 2 Oak Crest Institute of Science, Monrovia, CA 91016, USA; c.buser@oak-crest.org; 3 Norris Comprehensive Cancer Center, University of Southern California, Los Angeles, CA 90033, USA; kim.luhen@med.usc.edu (K.P.L.); Diane.DaSilva@med.usc.edu (D.M.D.S.); 4 Department of Obstetrics & Gynecology, University of Southern California, Los Angeles, CA 90033, USA

**Keywords:** anxA2, Birbeck granules, Langerhans cell, A2t

## Abstract

Langerhans cells (LC) are the resident antigen presenting cells of the mucosal epithelium and play an essential role in initiating immune responses. LC are the only cells in the body to contain Birbeck granules (BG), which are unique cytoplasmic organelles comprised of c-type lectin langerin. Studies of BG have historically focused on morphological characterizations, but BG have also been implicated in viral antigen processing which suggests that they can serve a function in antiviral immunity. This study focused on investigating proteins that could be involved in BG formation to further characterize their structure using transmission electron microscopy (TEM). Here, we report a critical role for the protein annexin A2 (anxA2) in the proper formation of BG structures. When anxA2 expression is downregulated, langerin expression decreases, cytoplasmic BG are nearly ablated, and the presence of malformed BG-like structures increases. Furthermore, in the absence of anxA2, we found langerin was no longer localized to BG or BG-like structures. Taken together, these results indicate an essential role for anxA2 in facilitating the proper formation of BG.

## 1. Introduction

Birbeck granules (BG) are cytoplasmic organelles which resemble tennis rackets in two-dimensional (2D) cross-sections. Since their discovery nearly 60 years ago, BG have largely remained elusive in derivation, composition, and function [1]. BG exist in a wide variety of morphologies when imaged through transmission electron microscopy (TEM), however, characteristic images typically contain a translucent “head” portion attached to a “rod” containing 5 to 10 nm linear striations through its center [2]. Three-dimensionally, BG “rods” are composed of two adjacent, superimposed zippered membranes forming a flat, circular disk or cytomembrane sandwiching structure (CMS) connected to the “head”, a vesicular lobe on the outer edge [3,4].

BG are the hallmark structures found in Langerhans cells (LC), which are the antigen presenting cells (APC) of the mucosal epithelium [5]. LC are highly specialized cells of the innate immune system which have the primary function of sampling their environment for foreign antigens [6]. Langerin (CD207), a type-II transmembrane c-type lectin, is the primary protein comprising BG and is a requisite for formation [7,8]. Collective evidence has demonstrated a function for langerin and BG in antigen binding, uptake, and processing through a nonclassical pathway [9,10,11,12]. In this process, langerin acts as a cell surface pattern recognition receptor (PRR) and is trafficked through the endosomal recycling compartment (ERC) under the control of Rab11a where it accumulates in recycling endosomes (RE) [13,14,15,16]. Once a critical intracellular langerin concentration is reached in the RE, a budding event facilitated by the Rab11a/myosin Vb/Rab11-FIP2 complex induces BG formation through membrane superimposition and a zippering of langerin interactions at the carbohydrate recognition domains (CRD) [4,5,7,17]. Cytoplasmic BG are proposed to process antigens for presentation via CD1a, an MHC-class I-like protein, to initiate the adaptive immune response through T cell activation [18].

Once a foreign antigen is recognized, LC undergo phenotypic and functional changes to become mature. This is characterized by an upregulation of co-stimulatory molecules such as CD80 and CD86, secretion of proinflammatory cytokines, and chemokine-mediated migration to lymph nodes where antigen-specific T cell priming occurs. LC are often the first immune cells to be in contact with pathogens that target or must bypass the mucosal epithelium such as human immunodeficiency virus (HIV) and human papillomavirus (HPV). BG structures sequester HIV and prevent its dissemination through selective degradation [10]. When exposed to HPV capsids, LC fail to mount a proper immune response. There is delayed maturation, a reduced level of MHC surface expression, and little to no costimulatory signals, which results in improper T cell priming [19]. We previously reported that this manipulation of LC by HPV is facilitated by the interaction with the annexin A2 S100A10 heterotetramer (A2t) [20]. Further investigation showed that A2t was directly involved in infectious trafficking of HPV virions [21]. In exploring the role of A2t in HPV–LC interactions, we made the interesting observation that BG structures were impacted by modulation of A2t expression, warranting further investigation into the relationship between A2t and BG formation.

## 2. Materials and Methods

### 2.1. Cell Culture

The CD34+ human acute myeloid leukemia cell line, MUTZ-3, was gifted by Rik J. Scheper from the VU Medical Center in Amsterdam, The Netherlands. MUTZ-3 cells were cultured at a density of 2 × 10^5^ cells/mL in minimum essential media, Alpha 1X (MEMα) with Earle’s salts, ribonucleosides, deoxyribonucleosides, and L-glutamine (Corning, NY, USA) supplemented with 20% heat-inactivated human fetal bovine serum (Omega Scientific, Tarzana, CA, USA), 10% conditioned medium from the renal carcinoma cell line 5637, and 50 μM 2-mercaptoethanol (Thermo Fisher, Carlsbad, CA, USA) at 37 °C with 5% CO_2_. To induce a Langerhans cell phenotype, MUTZ-3 cells were seeded at a density of 1 × 10^5^ cells/mL and were cultured for 14 days in the medium conditions described above. On days 0, 4, and 8, the cells were treated with a cytokine regimen containing 100 ng/mL GM-CSF (Sanofi, Bridgewater, NJ, USA), 2.5 ng/mL TNFα (PeproTech, Rocky Hill, NJ, USA), and 10 ng/mL human TGFβ (Thermo Fisher). Medium was replenished on day 8 of differentiation.

To generate 5637 conditioned medium, 5637 cells were cultured in RMPI 1640, 1X with L-glutamine (Corning) supplemented with 10% heat-inactivated human fetal bovine serum (Omega Scientific), 50 μM 2-mercaptoethanol (Thermo Fisher), and 1X gentamycin (Lonza, Walkersville, MD, USA) at 37 °C with 5% CO_2_. At 80% confluency, cells were harvested, seeded at a density of 15 × 10^6^ in a 175 cm^2^ tissue culture flask, and were allowed to reach confluency overnight. The medium was replaced at 24 h and collected at 72 h post seeding.

The spontaneously immortalized keratinocyte cell line, HaCaT, was cultured in Dulbecco’s modification of Eagle’s medium (DMEM) with 4.5 g/L glucose, L-glutamine, and sodium pyruvate (Corning, 10-013CV, NY, USA) supplemented with 10% heat-inactivated human fetal bovine serum (Omega Scientific), and 1X gentamycin (Lonza, Walkersville, MD, USA) at 37 °C with 5% CO_2_. Cells were passaged at 80% confluency.

### 2.2. Transmission Electron Microscopy

Cells were pelleted and fixed in 2.5% glutaraldehyde. Cells were post fixed with 1% osmium tetroxide (Electron Microscopy Sciences, Hatfield, PA, USA) in deionized water for 1 h at room temperature. The pellets were treated with 1% uranyl acetate for 1 h and, then, dehydrated through a graded addition of ethanol for 5 min each. Finally, the samples were embedded in epoxy resin of Eponate, NMA, DDSA, and DMP-30. Then, the pellets were sectioned into 70 nm ultrathin sections. Samples were imaged using a Jeol 2000 transmission electron microscope (JEOL USA, Peabody, MA, USA).

### 2.3. Immunoelectron Microscopy

Cells were processed for immunogold labeling, as previously described [22,23,24]. They were high-pressure frozen in PBS containing 20% BSA (Millipore-Sigma, Burlington, MA, USA) using an EMPact2 with RTS (Leica Microsystems, Vienna, Austria). Freeze-substitution was performed in acetone containing 0.1% uranyl acetate and 2% water in a Leica AFS2 (Leica Microsystems, Vienna, Austria). Then, cells were embedded in HM20 and UV polymerized at −50 °C for 24 h. Next, samples were sectioned into 70 nm sections, picked up on formvar-coated copper grids, and blocked for 10 min in blocking buffer (0.5% BSA in PBS). Primary goat anti-langerin (1:100; R&D Systems, Minneapolis, MN, USA), goat anti-anxA2 (1:1000; R&D Systems), and goat anti-S100A10 (1:1000; R&D Systems) were diluted in blocking buffer. After centrifugation at 14,000 rpm for 2 min, the supernatant was used to label the blocked sections for 30 min at RT, followed by five washes for 2 min each in 0.01% PBS Tween-20. Then, a rabbit anti-goat bridging antibody was diluted at 1:50 in blocking buffer. After centrifugation at 14,000 rpm for 2 min, the supernatant was used to label the sections for 30 min at RT, followed by five washes for 2 min each in 0.01% PBS Tween-20. Finally, 10 nm protein A gold (Electron Microscopy Sciences) was diluted to 1:50 in blocking buffer and used to label sections for 30 min at RT, followed by three washes for 2 min each with PBS and two washes for 2 min each with deionized water. The antibody-labeled sections were examined at 80 kV on a Morgagni 268 (FEI, Hillsboro OR, USA).

### 2.4. shRNA-Mediated Knockdown of A2/A2t

MUTZ-3 cells were transduced at a MOI of 5 with human annexin A2 shRNA or control shRNA lentiviral particles (Santa Cruz Biotechnology, Dallas, TX, USA) following the manufacturer’s protocol. Stably transduced cells were selected with puromycin. Then, A2/A2t knockdown MUTZ-3 cells were differentiated to M-LC, as described above. Knockdown of annexin A2 and S100A10 was, then, confirmed via Western blot.

### 2.5. Western Blot and Protein Quantification

Cells were lysed using RIPA buffer (Thermo Fisher) with Halt protease inhibitor (Thermo Fisher). Protein was quantified using a NanoDrop 2000 spectrophotometer (Thermo Fisher). Samples were run on a 10% Bis-Tris agarose gel with MES (Thermo Fisher). Gels were transferred using the iBlot2 mini system (Thermo Fisher) and were blocked in PBS Starting Block Blocking Buffer (Thermo Fisher) prior to primary antibody staining overnight at 4 °C with anti-anxA2 (BD Biosciences, San Jose, CA, USA), anti-S100A10 (BD Biosciences), anti-langerin (Cell Signaling Technology, Danvers, MA, USA), anti-beta-actin (Cell Signaling Technology), or GAPDH (Cell Signaling Technology). Secondary antibodies, anti-mouse IRDye 800CW (LI-COR, Lincoln, NE, USA), and anti-rabbit (H+L) Alexa Fluor 680 (Thermo Fisher), were added for 1 h at room temperature. Membranes were imaged using the Odyssey imaging system (LI-COR) and protein images were analyzed and quantified using Image Studio Lite software (version 5.2.3, LI-COR).

### 2.6. Flow Cytometry

Flow cytometry samples were collected using a Cytomics FC500 flow cytometer (Beckman Coulter, Brea, CA, USA) and data were analyzed using CXP software v2.2 (Beckman Coulter, Indianapolis, IN, USA). The following antibodies were purchased from BioLegend (San Diego, CA, USA): PE anti mouse/human CD207, PerCP/Cy5.5 anti-human CD1a, PE mouse IgG1, and PerCP/Cy5.5 mouse IgG1. For intracellular staining, cells were prepared according to the Affymetrix eBioscience protocol. Briefly, single cell suspensions were treated with IC Fixation Buffer, 1X Permeabilization Buffer, and the appropriate antibody, prior to being analyzed via flow cytometry.

### 2.7. Quantification of BG

Stereology was performed as described in Griffiths 1993 and was used to quantitate BG present in sections of 20 M-LC and 20 anxA2/A2t KD M-LC. Samples were blinded, and cells were chosen at random. Undifferentiated, aberrant, apoptotic cells, cells with no dendrites, and cells damaged during preparation (large cracks) were excluded. Each cell was imaged at a magnification of 22,000× (scale = 325 pixels/μm) and the images were stitched together using Fiji software package (v20170530, National Institutes of Health, Bethesda, MD, USA) to create whole-cell images. A lattice grid (grid distance of 1 μm) containing a systematic system of test points (points of intersection of test lines) was placed over each of the whole-cell micrographs. The number of points landing over BG and BG-like profiles was counted, as was the total number of points over the entire cytoplasm profile (Figure S3). To quantify differences in BG abundance, the following parameters were studied where P = points:

Points landing on BG per points landing on cytoplasm = P_BG_/P_cytoplasm;_
Points landing on BG-like structures per points landing on cytoplasm = P_BG-like_/P_cytoplasm._
A membrane structure was counted as BG-like if:

1. Sheet-shaped membrane = 
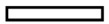

2. Or elongated sheet-shaped invagination of the plasma membrane;
3. Not ER (no ribosomes);
4. Not Golgi (not part of a Golgi stack);
5. Not vesicular.

A membrane was counted as BG if as BG-like but with a clear membrane-membrane boundary (“zipper”) anywhere within the membrane = 
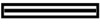


### 2.8. Statistical Analysis and Software Used

For EM images, statistical analysis was done using Stata v14.0 software (StataCorp, College Station, TX, USA) and verified by biostatistics consultation through the Southern California Clinical and Translational Science Institute (SC CTSI, Los Angeles, CA, USA). Data were plotted as box and whisker plots, and the Wilcoxon rank sum test was used to determine significance between groups. Significance was assigned to a value of *p* ≤ 0.05. For all other experiments, statistical analyses were performed using GraphPad Prism (v8, GraphPad Software, San Diego, CA, USA). Details for each individual experiment can be found in figure legends.

## 3. Results

### 3.1. MUTZ-3-Derived LC Are an Appropriate Model to Study BG Structure

In lieu of a commercially available cell line, LC studies often rely upon peripheral blood mononuclear cells (PBMC) isolated from human donors. However, using freshly isolated primary cells for structural studies of BG has several limitations, including donor heterogeneity. PBMC-derived LC cannot be maintained in a culture long term and are not amenable to gene manipulation. To work around these limitations, our study of BG structure utilized the immortalized MUTZ-3 cell line, which was generated from a CD34+ human acute myeloid leukemia [25]. Following a 14-day differentiation via cytokine regimen, langerin-expressing MUTZ-3-derived LC (M-LC) are generated with a consistent conversion rate of 30% to 40% as assessed by positive langerin (CD207) and CD1a expression and negative DC-SIGN expression (Figure S1A). These differentiated cells are phenotypically similar to primary human LC and have the same expression profile for langerin, CD1a, E-cadherin, HLA-DR, and other markers that are characteristic of LC [26,27]. M-LC are also functional in inducing anti-tumor T cell immunity [27], have similar transcription profiles to primary LC [28], and have been previously used to study BG sequestration of HIV [29]. Furthermore, M-LC have an abundance of BG [27], making it an ideal model system for our research question.

### 3.2. Immunogold Staining of Langerin in M-LC Gives Insight into Proper BG Structure Formation and Demonstrates a Novel BG Structure

The gold standard to study BG morphology is using TEM to capture 2D cross-sections of LC. The samples used for this process were prepared by high-pressure freezing and freeze-substitution, which provided excellent preservation of cellular structures and preserved epitopes for immunolabeling. We used a primary antibody, followed by a bridging rabbit anti-primary antibody and protein A bound to 10 nm gold particles to visualize the distribution of langerin in M-LC. The bridging antibody allowed us to standardize the labeling reaction across primary antibodies and also amplify the signal [30]. Langerin is a highly abundant, locally concentrated protein; gold particles found within images are localized to the cell and are rarely found in extracellular spaces (background = 0.22 gold/µm^2^), indicating the specificity of the labeling (Figure S1B).

**Figure 1:**
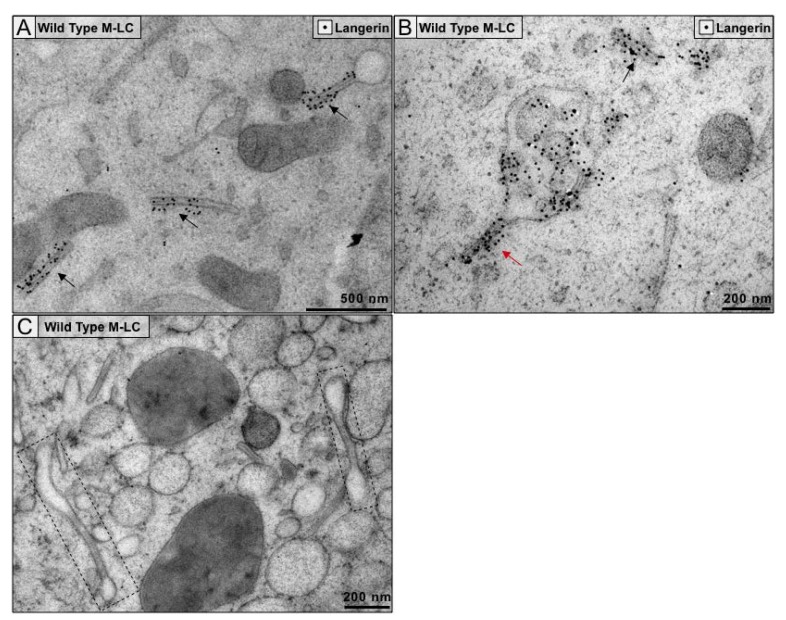
Birbeck granules in wild type MUTZ-derived langerhans cells (LC) (M-LC) have abundant langerin labeling localized to the cytomembrane sandwiching structure (CMS). (**A**) 10 nm gold particles (black arrows) show langerin labeling is localized to the CMS in Birbeck granules (BG); (**B**) Langerin labeling is contained within a cytoplasmic multivesicular endosome, likely a recycling endosome. Here, a BG CMS through langerin zippering from these stores is visible (red arrow); (**C**) BG structures containing multiple vesicular lobes on each end of the CMS (boxes). Images are representative of at least three biological replicates with a minimum of three grids each.

As others have reported, we observed abundant cytoplasmic BG and robust labeling of langerin localized to the BG rods (Figure 1A). The distribution of langerin labeling, as indicated by the arrows, highlights that langerin primarily localizes to the rod and is absent from the head portions of BG. These findings support previous observations that BG rod striations are formed through langerin interactions [17,31]. Langerin was also found at the cell surface and in invaginations at the plasma membrane, likely demonstrating endocytosis of surface langerin or recycling of langerin back to the surface (Figure S1C). As langerin trafficking and accumulation in the RE is a requisite to BG formation, it is not surprising that cytoplasmic vesicles containing langerin staining were also observed (Figure S1D).

BG have been established as subdomains of the ERC and bud from the RE through interactions between accumulated langerin and the Rab11a/myosin Vb/Rab11-FIP2 complex [5,16]. BG morphology is unique as compared with the RE in TEM; while RE are typically large vesicles (>500 nm diameter) containing internal membranes, BG are relatively smaller, consisting of a translucent vesicular lobe (<200 nm diameter) connected to a striated rod. For the first time, we captured the proposed BG budding event, from an RE containing intracellular langerin stores (Figure 1B). In this cross-section, distinct langerin labeling and membrane zippering show the formation of a BG budding from the RE. This vesicle is likely an RE, due to its size, the presence of internal membranes, and labeling of accumulated langerin. From this image, it appears that BG formation begins with langerin interactions creating the CMS as the head portion is not apparent and the budding rod is still attached to the RE. These findings provide visual support of the collective evidence regarding langerin recycling, trafficking and accumulation, and its driving role in BG formation from the RE.

In addition to the typical tennis-racket shaped BG structures, we also observed BG that resembled dumbbells in these electron micrographs (Figure 1C). According to the current model, BG are disk-shaped structures with a spherical vesicle-like formation at one of the ends [3]. However, these dumbbells demonstrate that BG are composed of multiple lobules or vesicles connected to a central CMS. To further understand the three-dimensional (3D) morphology of these dumbbell-shaped BG, we imaged serial sections of M-LC (Figure S1E). The same connectivity between a BG-sheet and the RE was also visible in serial sections (Figure S1E, arrow in bottom left corner of −200 nm through +200 nm). As these structures have not been observed in primary LC, these dumbbells could be unique to the MUTZ-3 system. It is possible that these structures do exist in primary cells but have not yet been reported. It is noteworthy, however, that these dumbbell structures were seen frequently in M-LC, as represented in Figure 1C and Supplemental Figure S1E.

### 3.3. Neither Subunit of A2t Colocalizes to BG Structures

**Figure 2:**
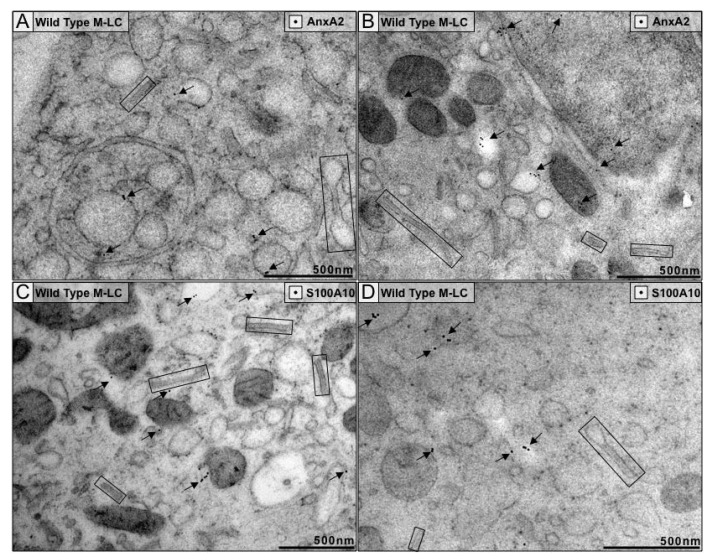
Neither subunit of the annexinA2 S100A10 heterotetramer (A2t) localize to BG in wild type M-LC. Immunoelectron microscopy with 10 nm gold particles was used to visualize the cellular distribution of anxA2 (**A**,**B**) and S100A10 (**C**,**D**). BG are boxed and gold labels are indicated by arrows. Images represent staining from tree independent biological replicates and a minimum of 3 grids.

A2t localization in M-LC was determined by immunolabeling using a polyclonal goat anti-annexin A2 antibody. This polyclonal antibody was raised against the full length of the anxA2 protein and allowed us to maximize the epitopes that could be recognized. AnxA2 binds membrane phospholipids, which was observed with gold labeling localized to the plasma membrane and membranes of organelles, as well as in the nucleus. We did not observe consistent localization of anxA2 labeling to BG (Figure 2A,B, Figure S2A,B). Since the anxA2 staining was not abundant, we verified the lack of A2t localizing to BG by also staining for S100A10. A polyclonal goat anti-S100A10 antibody raised against the full length of S100A10 was used to label the protein in M-LC. Similar to anxA2, S100A10 labeling was predominantly localized to non-BG structures, but some BG were labeled (Figure 2C,D and Figure S2C,D). The low levels of labeling only allowed us to speculate, based on the abundance of BG structures and the rare labeling of either anxA2 or S100A10 on them, that BG do not contain substantial amounts of A2t at steady state.

### 3.4. Knockdown of A2t Results in Reduced Expression and Non-BG Localization of Langerin

**Figure 3:**
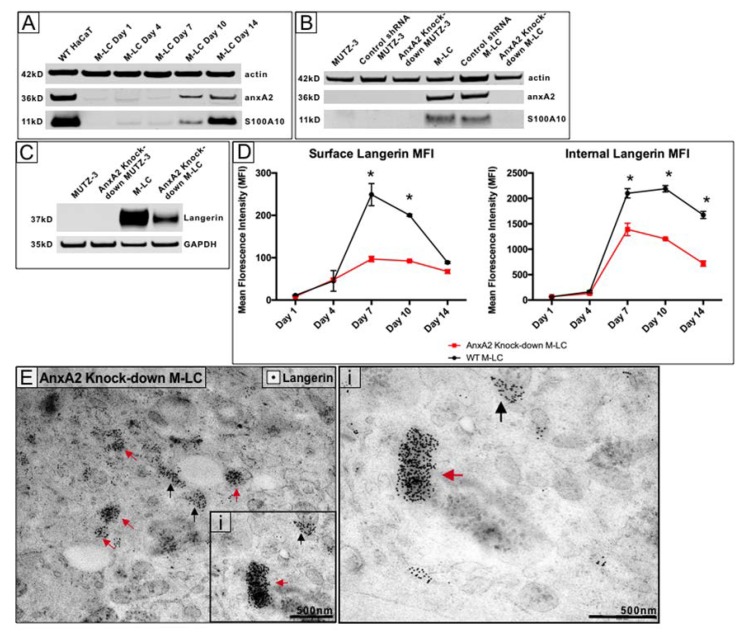
A2t knockdown in M-LC causes a decrease in langerin expression and aberrant cytoplasmic localization. (**A**) M-LC were collected throughout the 14-day differentiation for analysis of A2t expression via Western blot. The human keratinocyte cell line HaCaT were used as a positive control; (**B**) Western blot of MUTZ-3 and fully differentiated, day-14 M-LC show successful A2t knockdown in M-LC; (**C**) At day 14 of differentiation, langerin expression is decreased with A2t knockdown as compared with the WT M-LC; (**D**) Surface and internal langerin expression was assessed via flow cytometry in both wild type and A2t knockdown MUTZ-3 throughout differentiation. Data represents three biological replicates (*n* = 9) (* *p* < 0.05, paired Student’s *t*-test); (**E**) Black arrows indicate langerin labeling specific to BG and red arrows show abnormal, non-BG associated langerin clusters.

Because anx A2 is a key player in endosome biogenesis [32], next, we sought to investigate other ways that A2t could contribute to BG formation, such as mediating internal and surface langerin expression. Interestingly, predifferentiated MUTZ-3 do not express anxA2, however, M-LC do. M-LC lysates collected throughout the 14-day differentiation showed A2t expression beginning around day 10 (Figure 3A). AnxA2 was knocked-down in predifferentiated MUTZ-3 using a lentiviral sh RNA, causing S100A10 degradation as well (Figure 3B). We also found that overall langerin expression was decreased with anxA2 knockdown M-LC as compared with wild type (WT) M-LC (Figure 3C). Quantification of Western blot band densities are found in Supplemental Table S1.

In the steady state, langerin is regularly recycled from the cell surface through the ERC to form BG, and then trafficked back [5,14]. We sought to determine if the decreased langerin expression could be attributed to a recycling defect resulting in an altered distribution between intracellular and surface langerin. To examine this, we compared expression of surface and intracellular langerin in WT and anxA2 knockdown MUTZ-3 throughout the 14 days of M-LC differentiation via flow cytometry. Within anxA2 knockdown M-LC, surface langerin expression was significantly decreased on days 7 and 10 as compared with the WT M-LC (Figure 3D). Internal langerin expression was also significantly decreased in anxA2 knockdown M-LC on days 7, 10, and 14 (Figure 3D). These data indicate that anxA2 is involved in transport of langerin to the cell surface, as its absence significantly decreases surface langerin expression. It also suggests that langerin expression is dependent upon the expression of anxA2.

### 3.5. The Absence of A2t Results in Abnormal Cellular Distributionof Langerin and Incomplete BG Formation

To investigate changes with intracellular langerin distribution, we compared immunolabeled anxA2 in WT and knockdown M-LC. Intracellular langerin labeling in anxA2 knockdown M-LC visibly differed from WT M-LC (Figure 3E). In striking contrast to the robust rod-shaped labeling patterns seen in WT M-LC (Figure 1), langerin was found highly concentrated in clusters contained in vesicles throughout the cytosol, similar to the occasional endosomal labeling staining seen in WT M-LC. In the few discernable BG, langerin labeling was inconsistent and only partially localized to some BG structures (Figure 3E). Aberrant distribution of langerin observed with anxA2 knockdown suggests impaired or abnormal trafficking since langerin is no longer localized to BG structures and is instead contained in clusters throughout the cytosol.

**Figure 4:**
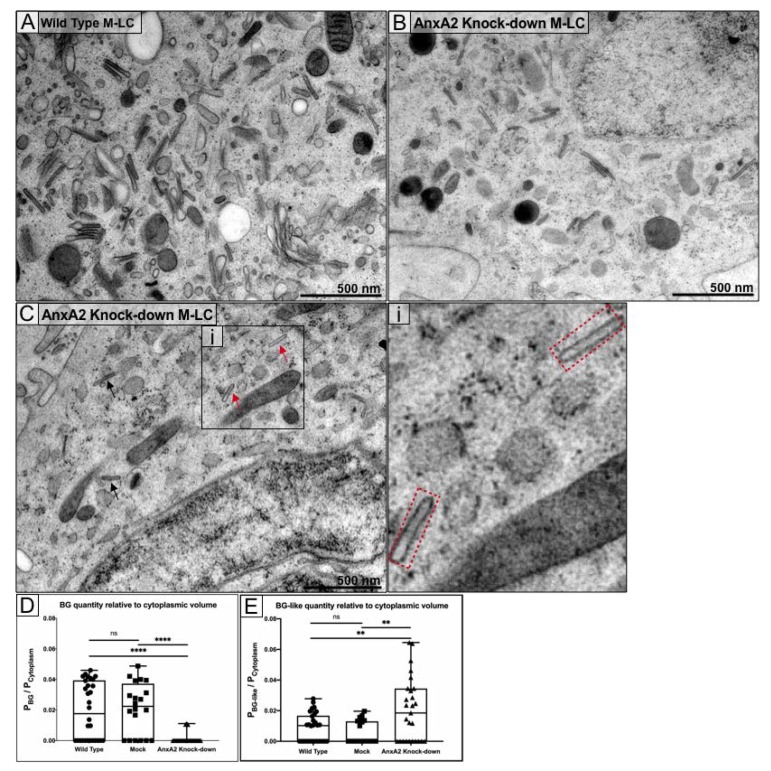
BG formation is significantly reduced in the absence of A2t. Wild type M-LC (**A**) contain an abundance of cytoplasmic BG as compared with A2t knockdown M-LC (**B**). At the same magnification, the relative abundance of cytoplasmic BGin WT and A2t knockdown M-LC is compared; (**C**) BG-like structures (red arrows) were observed in anxA2 knockdown M-LC and the missing striation through the center of the CMS is visualized (red box, insert i); (**D**,**E**) Stereology was used to analyze images for BG/BG-like quantification in wild type, mock-infected, and anxA2 knockdown M-LC. A 1 μm grid was overlaid to quantify cytoplasmic volume (Pcytoplasm) and BG abundance (PBG). Each point represents a single cell. Box and whisker plots show minimum, 1st quartile, median, 3rd quartile, and maximum values for each of the groups. ** *p* < 0.01, **** *p* < 0.0001, one-way ANOVA followed by Tukey’s multiple comparisons test. Data are representative of two biological replicates.

The most significant effect of anxA2 knockdown on M-LC was the near-disappearance of proper BG formation (Figure 4A,B). In addition to the decreased BG abundance, most BG found were incomplete as they were missing the central rod striations and had fewer misshapen heads but retained the unique flattened dimensions of BG rods (Figure 4C). We termed these abnormal structures as “BG-like”. To quantify the effect of anxA2 knockdown on BG formation, we compared WT and anxA2 knockdown M-LC using stereology [30], stitching together whole-cell cross-sections from individual TEM micrographs taken at 22,000×. Randomly chosen cells from two biological replicates were imaged and analyzed in 20 mock-infected, 30 wild type, and 27 anxA2 knockdown M-LC. BG and BG-like structures were quantified by overlaying 1 µm spaced grid on the cell and counting the grid intersection points that fall either on BG (PBG and PBG-like) or in the cytoplasm (Pcytoplasm) (Figure S3). The ratio of BG to cytoplasm in anxA2 knockdown M-LC was significantly decreased (*p* < 0.0001) as compared with wild type M-LC (Figure 4D). BG-like occurrence in anxA2 knockdown was also significantly increased as compared with wild type M-LC (*p* = 0.0056) (Figure 4E).

## 4. Discussion

In this body of work, we have described an essential role for A2t in BG formation. Annexins have been shown to have diverse cellular functions and are known for their membrane remodeling capabilities [33]. A2t itself is a multifunctional protein comprised of two monomeric annexin A2 (anxA2) subunits bridged by an S100A10 dimer and has been described in cellular trafficking events such as promoting actin remodeling and facilitating endosomal transport [32,33]. Interestingly, anxA2 is found specifically within the membranes of Rab11a^+^ RE and proper intracellular distribution of RE is dependent upon anxA2 [34,35]. Using the transferrin receptor to model the ERC, A2t depletion drastically alters the shape and distribution of recycling endosomes [35]. The literature demonstrates A2t regulation of RE morphology and cellular distribution, processes which are also utilized in LC for the formation of BG.

Given the relatively unknown processes regulating BG formation, we cannot definitively describe how A2t is involved in this process. The loss of proper BG formation in the absence of A2t shows a defect in the ability of langerin to interact to form CMS and ultimately BG. We speculate, based upon the literature and the observed BG structural abnormalities in this study, that A2t acts in conjunction with Rab11a at the RE to promote langerin interactions and the formation of BG. Despite our efforts, we were not able to discern any localization of A2t in BG or a direct functional role that A2t plays in mediating langerin trafficking and BG formation. However, since A2t often acts as a scaffolding protein, it could facilitate BG structural formation indirectly. As such, we would not necessarily expect to find it localized to BG via IEM imaging following formation in a steady state.

On the basis of the data presented in this study, the loss of BG and the formation of BG-like structures can be attributed to anxA2 deficiency. In the absence of A2t, langerin trafficking through the ERC is disrupted, leading to langerin clustering and accumulation throughout the cytosol and a significant loss of BG formation. For further elucidation of the role of anxA2 in BG formation, additional studies would be needed to investigate whether anxA2 physically interacts with langerin or Rab11a. Future mechanistic studies could also involve time course experiments studying changes in trafficking of langerin in the absence of anxA2. Overall, we have highlighted A2t as a new, previously unidentified, player in Langerhans cell BG formation and added to the foundational knowledge of BG for future studies that focus on characterizing their biogenesis, structures, and functions.

## Supplementary Materials

The following are available online at https://www.mdpi.com/2073-4409/9/4/974/s1, Figure S1: MUTZ-3 derived LC is an acceptable model to study BG morphology and langerin localization using IEM, Figure S2: Supplemental images to Figure 2 of AnxA2 and S100A10 labeling in WT M-LC, Table S1: Western blot band quantifications. Figure S3: Whole-cell composite example for quantifying BG an BG-like structures using stereology.
