# Supplementary material for: The Essential Role of anxA2 in Langerhans Cell Birbeck Granules Formation
Source: Cells. 2020 Apr 15;9(4):974. doi: 10.3390/cells9040974 (PMC7227008; doi:10.3390/cells9040974)

Supplementary Figure 1 – MUTZ-3 derived LC is an acceptable model to study BG morphology and langerin localization using immuno-electron microscopy. (a) Flow cytometry characterization of fully differentiated M-LC stained for langerin, CD1a, and DC-SIGN. Around 30% of M-LC are double positive for Langerin and CD1a and are negative for DC-SIGN (red boxes). **(b)** Quantification of background labeling for langerin in M-LC. Six randomly chosen non-cytoplasmic areas from grids immuno-labeled for langerin were analyzed for the number of gold particles per extracellular space. **(c)** EM image at the M-LC plasma membrane showing langerin at an invagination (arrows). **(d)** Langerin immunolabeling in M-LC showing a langerin-containing endosome near the plasma membrane. **(e)** Serial images of M-LC demonstrating the morphology of BG containing multiple vesicular lobes attached to the CMS (arrows). Each image represents a subsequent section of the same cell and begins at the anterior most section (-250nm), moving distally in 50nm increments to the posterior of the cell (+200nm). Images are representative of three independent experiments and at least three grids.

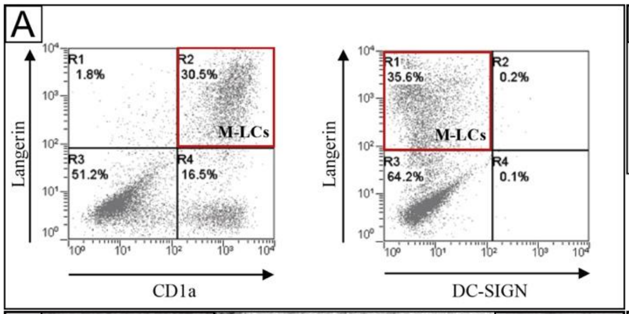

B

Cell	10nm protein A gold	Size (μm^2)	Particles/μm^2
1	2	12.41	0.1612
2	1	12.41	0.0806
3	5	12.41	0.4029
4	2	12.41	0.1612
5	3	12.41	0.2417
6	4	12.41	0.3223
Average	2.8	12.41	0.2283

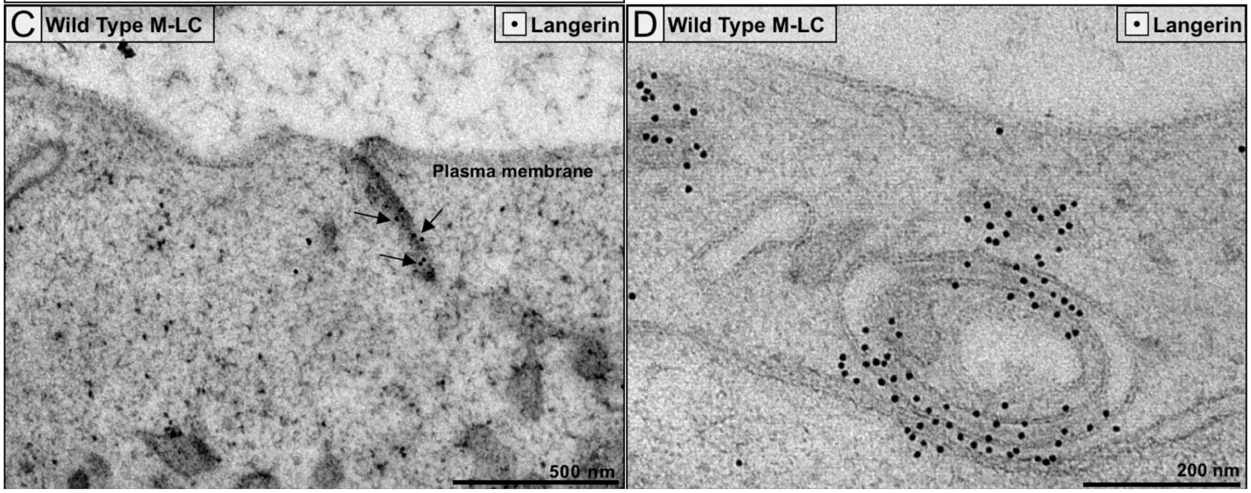

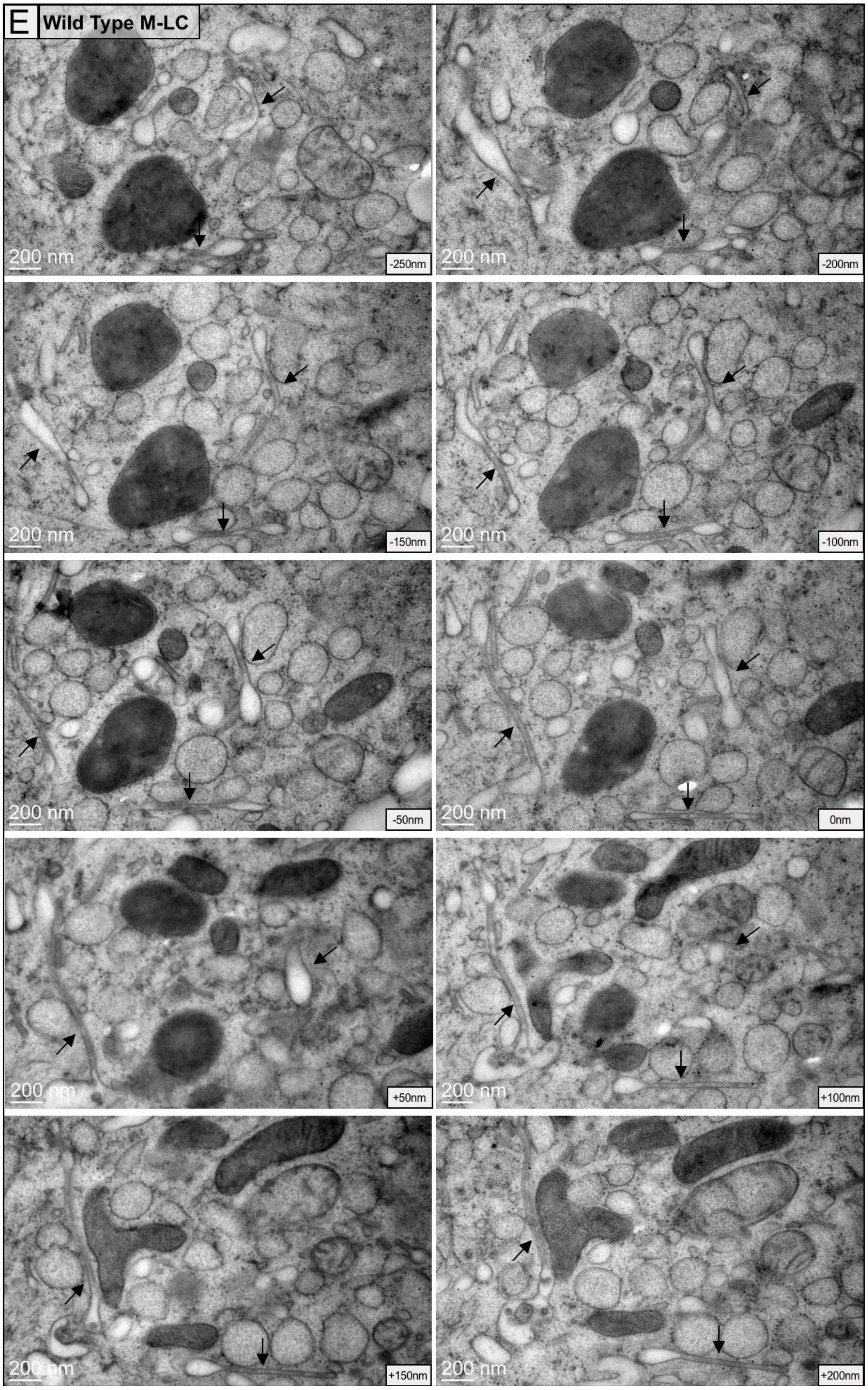

E
Wild Type M-LC
200 nm
-250nm
200 nm
-200nm
200 nm
-150nm
200 nm
-100nm
200 nm
-50nm
200 nm
0nm
200 nm
+50nm
200 nm
+100nm
200 nm
+150nm
200 nm
+200nm

Supplementary Figure 2 – Supplemental images to figure 2 of AnxA2 and S100A10 labeling in WT M-LC. (a, b) Immunolabeling for anxA2 is seen in many expected locations throughout the cytosol (arrows) but not in BG (boxed). **(c, d)** Similarly, S100A10 staining is rarely seen in cytoplasmic BG (boxes) but was found to have a similar cytoplasmic distribution as anxA2, indicating the accuracy of labeling for each protein. Images are representative of three independent experiments and at least five grids.

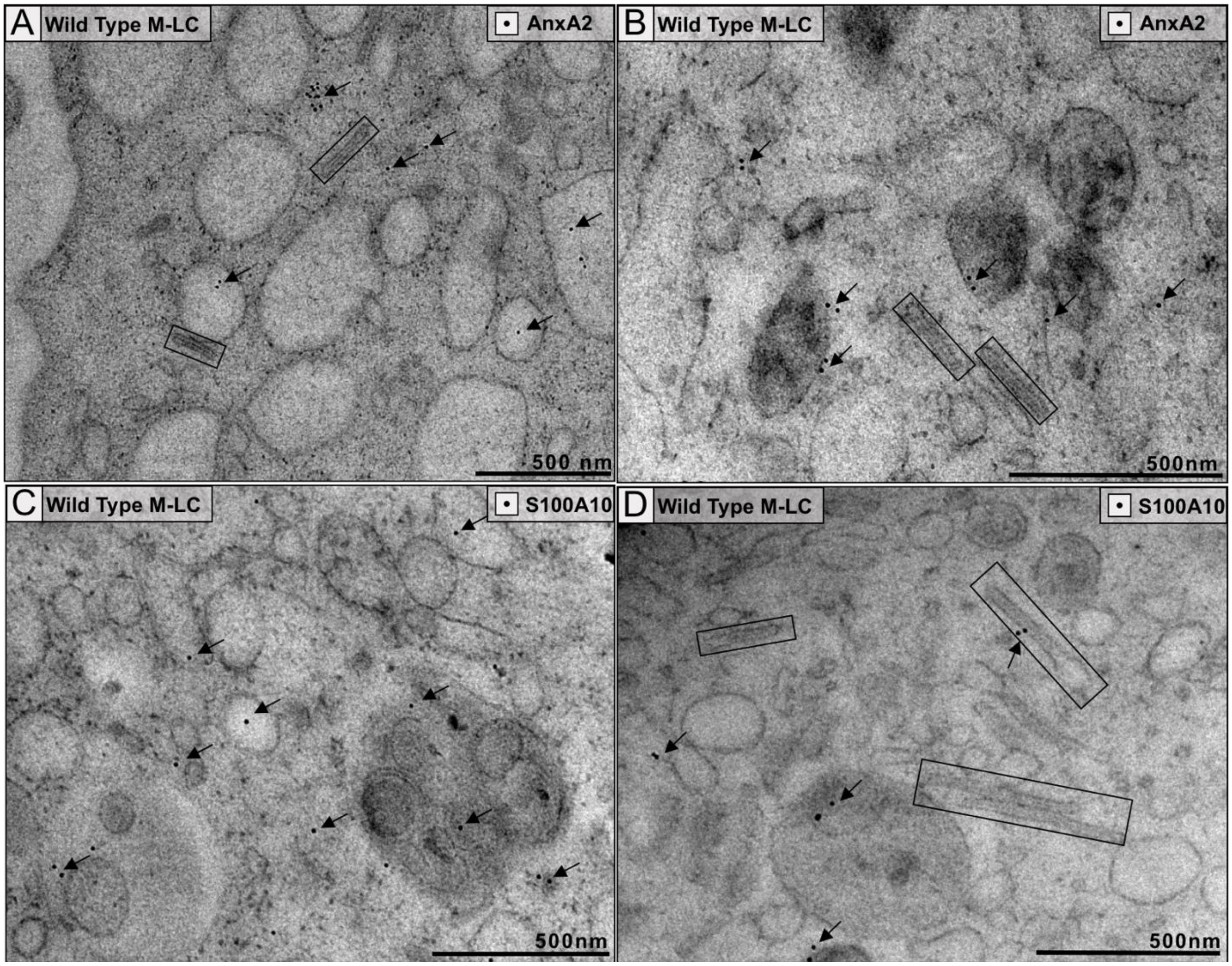

Supplementary Table 1 – Quantitation of protein bands in Figure 3A, B, and C, normalized to housekeeping protein loading controls. Band intensities were quantified in ImageStudioLite and normalized to the loading control for each.

	Lanes					
**Figure 3A**	**HaCaT**	**M-LC D1**	**M-LC D4**	**M-LC D7**	**M-LC D10**	**M-LC D14**
Relative band intensity of anxA2 to M-LC D1	2642.02	1.00	1.46	3.19	633.28	1272.51
Relative band intensity of S100A10 to M-LC D1	4815.26	1.00	6.00	6.56	449.22	3338.09

	Lanes					
**Figure 3B**	**MUTZ-3**	**Control shRNA MUTZ-3**	**AnxA2 KD MUTZ-3**	**WT M-LC**	**Control shRNA M-LC**	**AnxA2 KD M-LC**
Relative band intensity of anxA2 to WT MUTZ-3	1.00	1.40	1.01	30.96	45.30	0.80
Relative band intensity of S100A10 to WT MUTZ-3	1.00	1.19	0.97	240.98	267.98	9.46

	Lanes			
**Figure 3C**	**MUTZ-3**	**AnxA2 KD MUTZ-3**	**WT M-LC**	**AnxA2 KD M-LC**
Relative band intensity of langerin to WT MUTZ-3	1.00	0.64	8927.34	1175.70

Supplementary Figure 3 – Whole-cell composite example for quantifying BG an BG-like structures using stereology. Example of a composite whole-cell micrograph overlaid with a 1μm grid. Red circles indicate the grid intersections falling within the cytoplasm ($P_{Cytoplasm}$). Grid points (red circles) landing over BG (P_{BG}) and BG-like structures ($P_{BG\text{-}like}$) was quantified and compared to the cytoplasmic volume.

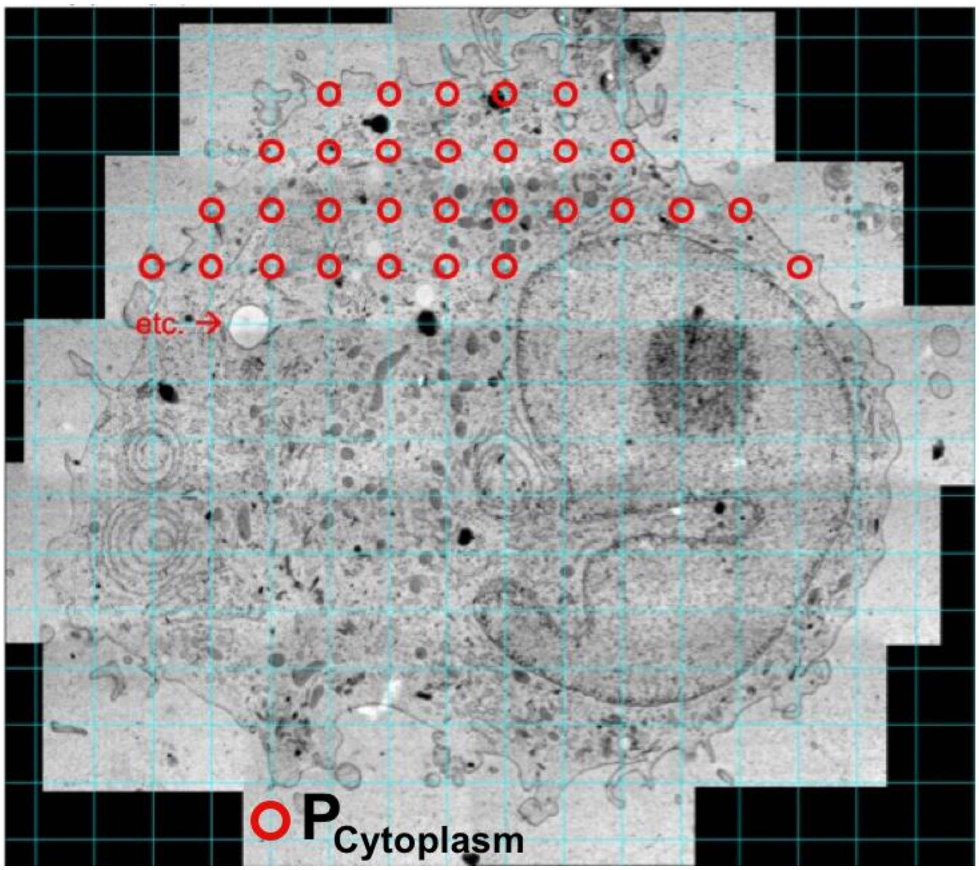

Supplement: Supplementary file 1 [file cells-09-00974-s001.pdf]
